# Buttock Augmentation with Ultrasonic Liposuction and Ultrasound-Guided Fat Grafting: A Retrospective Analysis Based on 185 Patients

**DOI:** 10.3390/jcm13061526

**Published:** 2024-03-07

**Authors:** Ahmed Elsaftawy, Patryk Ostrowski, Michał Bonczar, Mateusz Stolarski, Kamil Gabryszuk, Tomasz Bonczar

**Affiliations:** 1 Chiroplastica—Lower Silesian Centre of Hand and Aesthetic Surgery, 54-117 Wroclaw, Poland; 2 Department of Anatomy, Jagiellonian University Medical College Cracow, 33-332 Cracow, Poland; 3 Youthoria, Youth Research Organization, 30-363 Cracow, Poland

**Keywords:** buttock augmentation, liposuction, fat grafting, plastic surgery, aesthetic surgery

## Abstract

**Background:** The popularity of gluteal augmentation has surged in recent decades, primarily due to satisfactory aesthetic results. **Objectives:** The primary goal of this retrospective analysis was to present the key characteristics of a large patient cohort from Europe, comprising 185 female patients who underwent gluteal augmentation with ultrasound-guided fat grafting. **Methods:** A retrospective analysis was conducted on 185 female patients who underwent gluteal augmentation with ultrasonic liposuction and fat grafting from February 2020 to July 2023. The procedures were performed in accordance with the latest safety recommendations. **Results:** Information concerning the patient demographic, volume of fat graft, and complications was analyzed. Additionally, patient satisfaction was evaluated through a questionnaire. The mean Body Mass Index (BMI) of the patients was 23.00 kg/m^2^ (SD = 2.62). Furthermore, the volume of transferred fat graft ranged from 500.00 mL to 800.00 mL, with a mean of 596.75 mL (SD = 67.29). The most frequent complication was the formation of seroma (5.41%). All complications were treated successfully. **Conclusions:** Using ultrasonic liposuction and ultrasound-guided fat grafting to enhance buttock contours is a reliable technique. Nevertheless, it is crucial to exert caution and take all necessary steps to reduce the risk of potentially life-threatening complications.

## 1. Introduction

Gluteal augmentation with fat grafting has increased in popularity over the last decades [1]. This procedure is designed to meet the needs of patients who wish to augment their buttocks by increasing volume and achieving a curvier appearance. Gonalez and Spina [2] first presented the technique of gluteal augmentation using fat grafts in 1986. Two years later, Matsudo and Toledo [3] published the first English article describing their experience with fat augmentation and gluteoplasty. Since then, numerous techniques of buttock augmentation with fat grafts have been developed and showcased. These techniques involve injecting the fat graft in various planes, such as intramuscularly or subcutaneously, each presenting its own set of advantages and disadvantages [4].

Although gluteal augmentation with fat grafting is among the fastest-growing aesthetic procedures in the United States and around the world, there have been a significant number of reports of fatal and nonfatal complications [5]. The spectrum of complications associated with this procedure ranges from relatively mild, such as intermittent discomfort or seroma formation, to potentially life-threatening conditions like fat emboli and septic shock [4,6]. Nevertheless, previous reports have concluded that gluteal augmentation using autologous fat grafting leads to fewer postoperative complications than implants and allows greater contouring of the buttocks and the surrounding regions [7]. Although numerous reports have shed light on this procedure, most originate from South or North America, leaving a scarcity of data from Europe [4]. Therefore, the primary goal of this retrospective analysis was to present the key characteristics of a large patient cohort from Poland, comprising 185 female patients who underwent gluteal augmentation with ultrasound-guided fat grafting. Additionally, we will discuss the complications associated with this procedure and explore methods to reduce their occurrence, particularly those posing life-threatening risks.

## 2. Materials and Methods

A retrospective investigation of the clinical outcomes of gluteal augmentation with ultrasound-assisted liposuction and ultrasound-guided fat grafting was conducted. The medical records of 185 consecutive patients who underwent the aforementioned procedure between February 2020 and July 2023 were analyzed. The present analysis adhered to the ethical guidelines specified in the Helsinki Protocol, exemplifying a firm dedication to upholding the tenets of autonomy, beneficence, and non-maleficence. This research relied on retrospective data extracted from medical records, guaranteeing meticulous attention to safeguarding the confidentiality and anonymity of the participants. All patients provided informed consent for the surgery and voluntary participation in the study. The consent for both was collected prospectively prior to the surgical procedure.

Before the operation, all patients underwent consultation and a qualification procedure. The following inclusion criteria were enrolled: (1) individuals dissatisfied with their current buttock aesthetics; (2) gluteal skin devoid of any pathological alterations; (3) no compelling contraindications, especially regarding the circulatory, respiratory, and nervous systems; (4) a body fat percentage within a reasonable range of 20–30%; and (5) a Body Mass Index between 20 and 30 kg/m^2^. Patients who did not meet the inclusion criteria were excluded from both the surgical procedure and the present study. Furthermore, additional exclusion criteria were also employed: (1) chronic pain in the gluteal area; (2) current spinal disk herniation.

**Figure 1:**
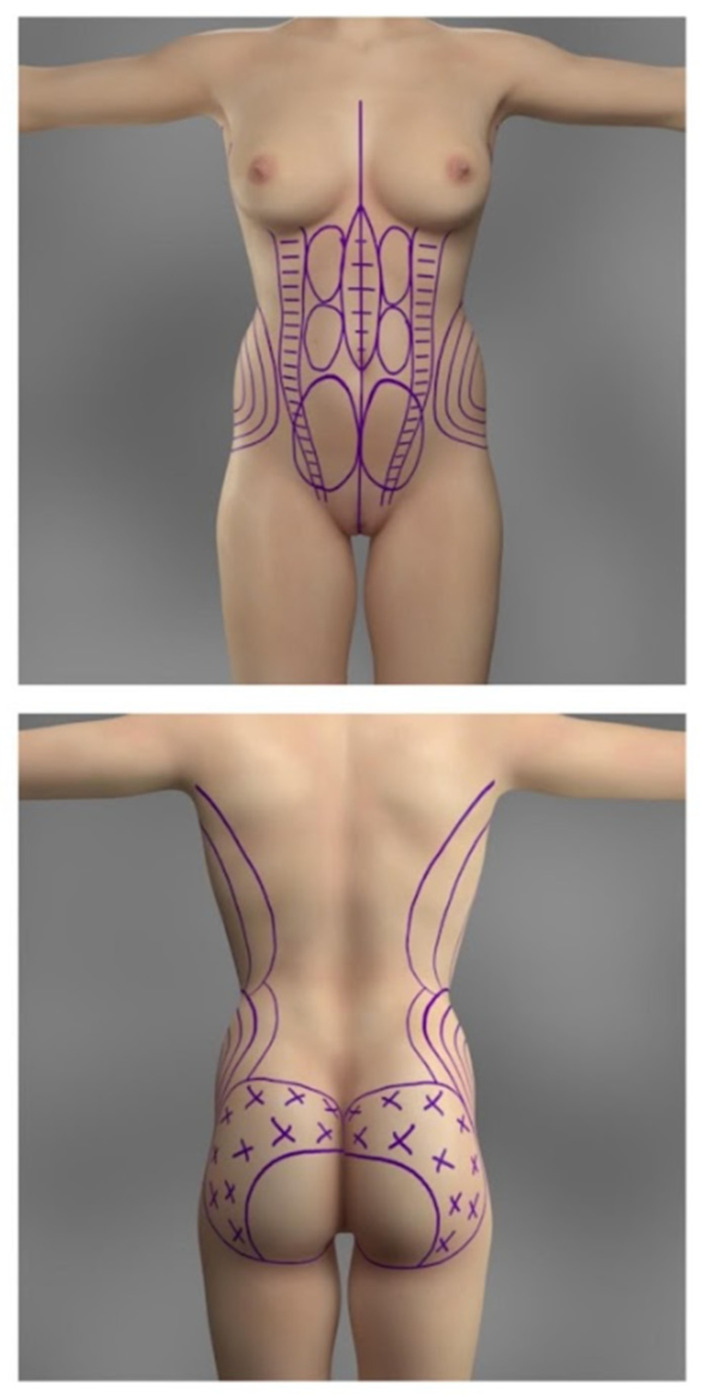
Preoperative markings demonstrate areas of fat harvesting and injection.

**Figure 2:**
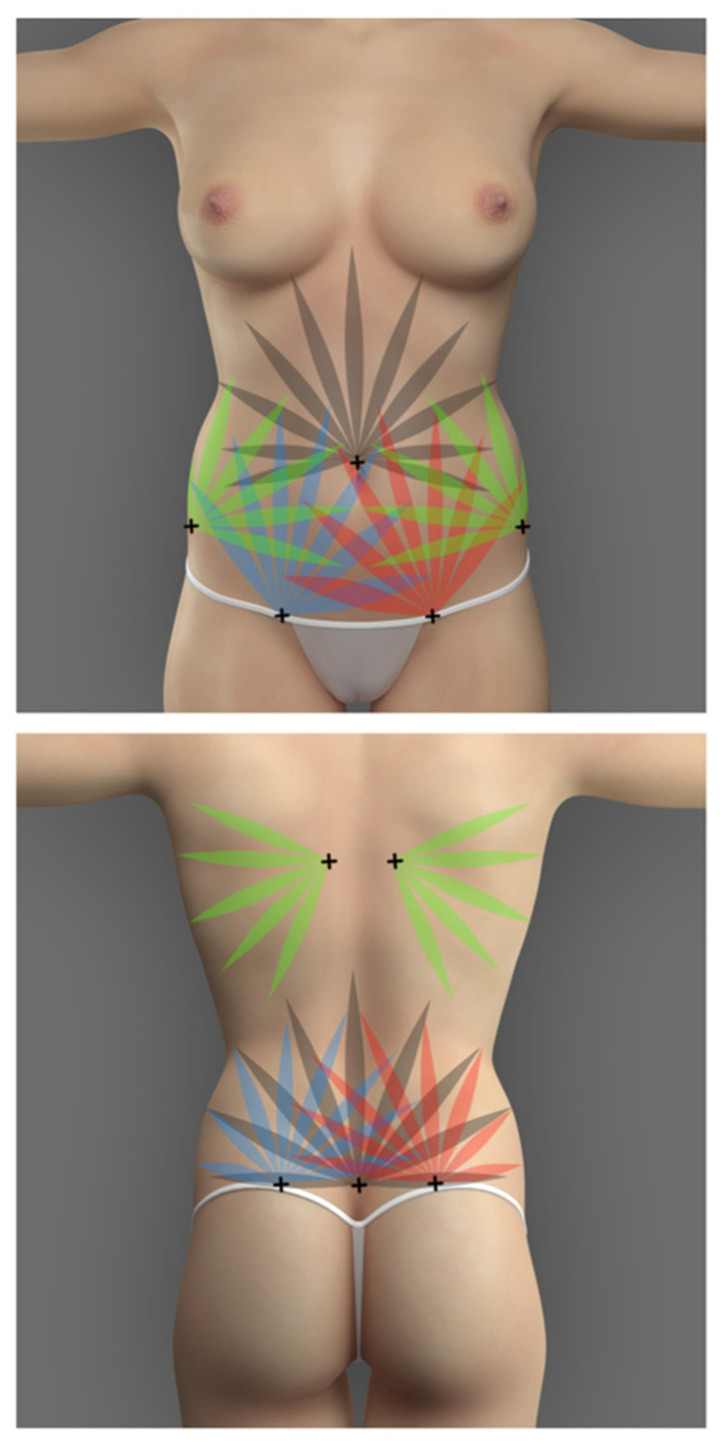
Locations of port placements (crosses) and the direction of liposuctioning from each of the ports.

The potential areas for liposuction and fat grafting were marked preoperatively and are demonstrated in Figure 1. The most frequent body areas to be suctioned include the lower and upper abdomen, the flanks, the upper torso, and, rarely, the thighs. However, acknowledging the variability of the choice of suctioned areas is crucial, as every patient is different and has different fat distributions, proportions, and personal expectations. Thus, the port placement depends on the areas planned to be liposuctioned. Most commonly, the port incisions are marked as follows: bilateral incisions lateral to the pubic symphysis in the groin (for the liposuction of the anterior lower abdomen), bilateral incisions superolaterally to the anterior superior iliac spines (for the liposuction of the lower flanks and the anterior abdomen), one incision above the umbilicus (for the liposuction of the upper abdomen), bilateral incisions between the scapulae at the level of the bra-line (for the liposuction of upper flanks), bilateral incisions over the superior posterior iliac spine (for the liposuction of the lower back and flanks), and lastly, one at the superior apex of intergluteal cleft (for the liposuction of the lower back). The port placements are depicted in Figure 2. The buttock augmentation was performed using general anesthesia. The technique described in the present study is demonstrated in the Supplementary Video (Video S1 in the Supplementary Materials).

The antibiotic regimen employed during the perioperative phase involved administering preventive treatment using third-generation cephalosporins (specifically ceftriaxone) at a dose of 2 g intravenously. In cases where patients had an allergy to penicillin, an alternative approach involved using 900 mg of clindamycin. This proactive use of antibiotics primarily targets the prevention of infections caused by strains of *Staphylococcus aureus* and *Streptococci*.

The procedure starts with injecting an infiltration solution of 0.9% 1 L saline and epinephrine (1:500,000), 20 mL 2% lignocaine, and 500 mg of tranexamic acid. The main goal of administering the said solution is to infiltrate the fat, aid in transmitting the ultrasonic waves to the fat, decrease the risk of heat-induced injury, and decrease blood loss. Moreover, the 2% lignocaine may aid in immediate postoperative pain management.

The ultrasonic liposuction is performed with the help of the VASER device (Sound Surgical Technologies, Louisville, CO, USA) that breaks down the adipose tissue before the suctioning. The transmission mode of the ultrasonic waves is set to “continuous”, with wave amplitude settings ranging from 70 to 80%. The probes were placed in the aforementioned port openings and secured externally to decrease the risk of mechanical or heat-induced damage to the superficial tissues surrounding the incisions. In the anterior abdomen, the depth of the ultrasonic liposuction varies in different areas. This is also depicted in Figure 1; the three bilateral circles lateral to linea alba represent the area where only the deep adipose tissue is broken down, leaving the superficial subcutaneous tissue alone. The superficial subcutaneous tissue is left alone in the area lateral to the linea alba, to decrease the risk of potential excessive skin laxity. The subcutaneous and deep adipose tissue might get broken down at the lateral edges of the rectus abdominis muscle (two bilateral columns with horizontal lines) and directly over the linea alba (oval circle with horizontal lines). At the upper and lower flanks, mostly deep adipose tissue is taken, and some is subcutaneous. The time of ultrasonic wave projection is shorter (under 3 min) in areas with less fat (for reference, an area smaller than the palm of a hand). This time may even be shorter if the patient has less collagen tissue in the fat, making it less resistant to the forces of the probe. Exaggerated liposuction in such patients may lead to areas of loose and saggy skin. The ultrasonic suctioning is performed in a crisscross manner towards the bilateral port’s location to get an even and aesthetic result. Preoperative and intraoperative assessment with ultrasound imaging was performed to ensure no significant pathological changes in the gluteal anatomy; however, the percentage of fat attachment was not assessed. The direction of liposuctioning from each port is illustrated in Figure 2. After the ultrasonic breakdown of the fat in the selected regions of the patient, a 3.5 mm cannula is used to harvest the fat.

The harvested fat is transferred to a sterile, closed container and decanted for 30 min to separate the adipose cells from the liquid. Next, the liquid is emptied, leaving only the fat in the container. The fat is then aspirated into 60 mL syringes and centrifuged to separate the remaining fluid from the fat. Moreover, venous blood is obtained from the patient, and platelet-rich plasma is prepared. Lastly, the isolated fat and the platelet-rich plasma are mixed in a 1:5 ratio, mainly to improve the survival of the fat graft [8]. This fat graft is finally ready for transfer to the gluteal region.

The injection process is performed with the patient in the prone position. The fat graft is transferred only to the subcutaneous layer due to the increased risk of severe complications associated with intramuscular fat injections [9]. To ensure safe injection, intraoperative ultrasound was utilized. The areas of injection are shown in Figure 1. The fat graft is injected with a large bore blunt 4.0 mm cannula, using a continual motion to prevent potential injection into a vessel and to ensure evenness of graft placement. Moreover, the injection is performed first in the lateral aspects of the buttocks or the trochanteric areas and, later, in the middle/medial regions. However, as depicted in Figure 1, the fat graft is not transferred into the inferomedial area of the gluteal region, mainly to prevent possible iatrogenic injury to crucial neurovascular structures and to decrease the risk of drooping of the inferior aspect of the buttocks [10,11]. The endpoint of the fat injection process was when the aesthetic goals and expectations of the patients were met.

The pain management protocol involved the following steps: administration of paracetamol at a dose of 500 mg, four times a day, alongside ketoprofen at 2 mg, taken twice daily, to act as an analgesic. In cases of heightened pain, tramadol was included in the prescription. Enoxaparin (40 mg) was given for 10 days as an anticoagulative treatment. The patients were recommended to avoid sitting or lying in the supine position postoperatively (ideally for up to two days). Moreover, the patients were advised not to engage in strenuous physical activities for a period of four weeks following the procedure. Additionally, the patient had to lay in a prone position postoperatively for up to two weeks. The postoperative evaluation was carried out by the operator performing the procedure. The first follow-up appointment occurred one week after the surgery. During this visit, the dressing covering the wounds was removed, and the incisions were carefully cleaned using a saline solution. The patients were instructed to wear compression garments for at least six weeks after the surgery to aid in sculpting the buttocks. We did not use drains, and hence, lymphatic massages were a crucial step of the postoperative care, helping to minimize fluid collection. The massages were implemented into the postoperative care five to seven days after the surgery and were subsequently performed two times a week for five weeks. Each patient was scheduled for follow-up appointments at six weeks, 12 weeks, and six months, respectively.

The complications in the present retrospective analysis included 10 cases of seroma formation, a case of abscess formation, and two cases of minor infections by incision sites for fat harvesting. Seroma formation occurred most commonly in the lower anterior abdomen, and all seromas were drained successfully. The abscess formed near the incision site for fat harvesting and was already opened during the initial investigation. It was drained and cleaned, and the pus was sent for laboratory analysis. The treatment consisted first of a course of broad-spectrum antibiotics registered intravenously (amoxicillin 400 mg two times daily and metronidazole 500 mg two times daily, for five days), and then specific antibiotics for the strain of bacteria. The two cases of minor infections were also observed during the first follow-up and were treated with the same regimen of intravenous broad-spectrum antibiotics as mentioned above. No cases of liponecrosis occurred in our patient cohort.

Patient satisfaction was evaluated through a brief survey distributed after the final follow-up session, which occurred six months post-procedure. The survey gauged the level of satisfaction among the patients concerning the general silhouette shape, the aesthetics of the abdomen, and the shape, size, and projection of the buttocks. Patient satisfaction was categorized into four groups: very dissatisfied, somewhat dissatisfied, somewhat satisfied, and very satisfied.

### Statistical Analysis

Statistical analysis was conducted using SATISTICA v13.1 software (StatSoft Inc., Tulsa, OK, USA). The frequencies and percentages were utilized to describe qualitative characteristics. The Shapiro–Wilk test was employed to evaluate the normal distribution. Quantitative attributes were described using medians and upper and lower quartiles (UQ and LQ, respectively), as well as means and standard deviations (SDs), contingent upon the confirmed normality of the dataset. Statistical significance was defined as *p*-values less than 0.05. Spearman’s rank correlation coefficient was employed to identify potential correlations among the parameters.

## 3. Results

### 3.1. Patient Characteristics

A retrospective analysis of the results of 185 female patients who underwent gluteal augmentation with ultrasound-assisted liposuction and fat grafting was performed. The age of the patients ranged from 22–56 years, with a mean age of 34.56 years (SD = 7.31). The weight of the patients ranged from 48.50 kg to 98.00 kg with a mean weight of 64.46 kg (SD = 8.22), whereas the height of the patients ranged from 152 cm to 188 cm with a mean height of 167.39 cm (SD = 5.82). The mean Body Mass Index (BMI) of the patients was 23.00 kg/m^2^ (SD = 2.62). All of the patient characteristics are presented in Table 1.

### 3.2. Fat Graft

**Figure 3:**
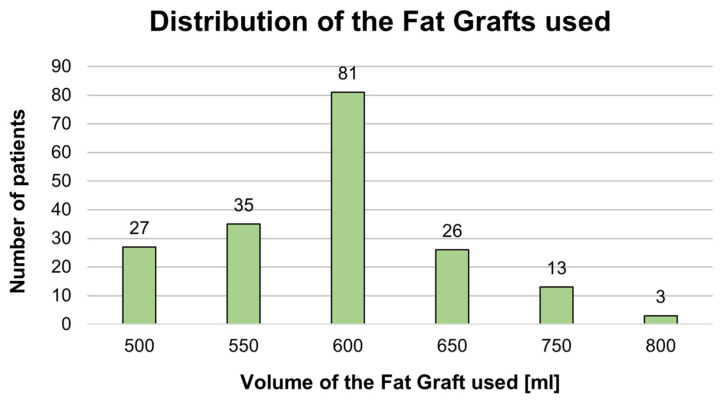
Diagram presenting the distribution of the fat grafts used in all patients.

The volume of fat transferred ranged from 500.00 mL to 800.00 mL, with a mean of 596.75 mL (SD = 67.29). In 27 (14.59%) of the patients, a 500 mL fat graft was used. In 35 (18.91%) of the patients, a 550 mL fat graft was used. In the majority of the patients (81 patients; 43.78%), a 600 mL fat graft was used. In 26 (14.05%) patients, a 650 mL fat graft was used. In 13 (7.03%) of the patients, a 750 mL fat graft was used. In three (1.62%) patients, an 800 mL fat graft was used. Detailed characteristics regarding the volume of the fat graft used are shown in Table 1 and Figure 3.

Spearman’s rank correlation analysis was performed in order to establish the possible correlations between the patient’s age, weight, height, and BMI and the volume of the fat graft used. The weight of the patients was found to statistically significantly correlate (*p* = 0.00) with the volume of the fat graft used (R = 0.55). The statistical correlations are demonstrated in Table 2.

### 3.3. Complications

**Figure 4:**
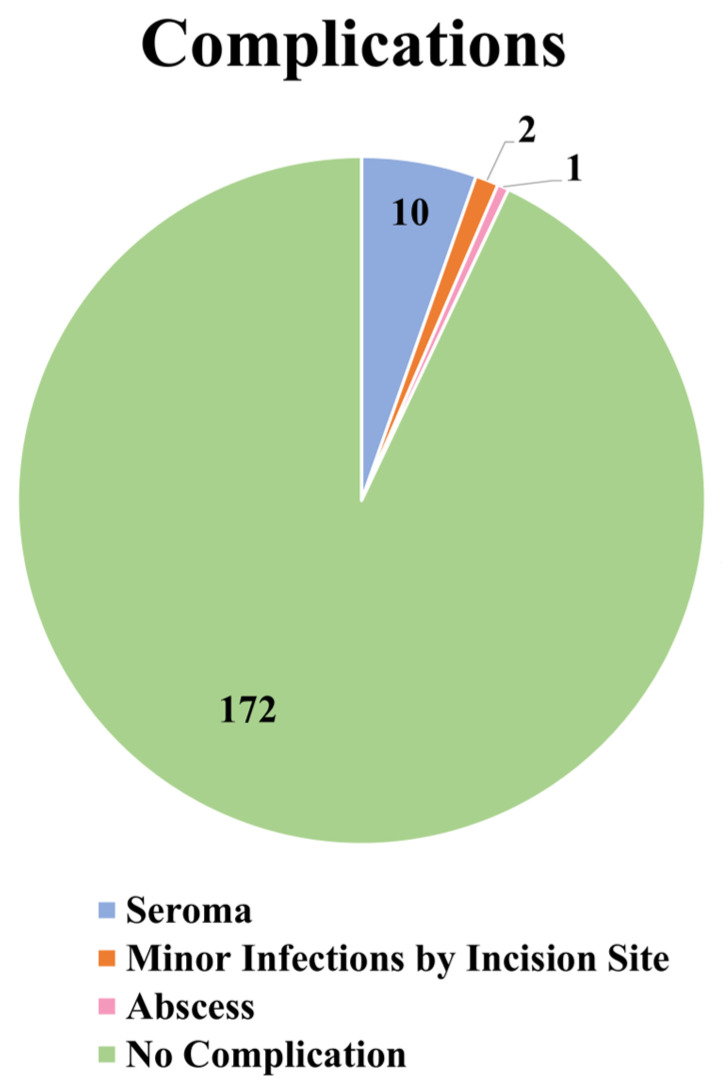
Pie chart demonstrating the complications.

The total complication rate in the present study was found to be 7.03% (*n* = 13). The complications were divided according to their severity. Twelve complications were classified as minor, whereas one was classified as major. The most common complication was seroma, which occurred in 10 of the 185 patients (5.41%). Minor infections by the incision site occurred in two patients (1.08%). The total rate of minor complications was 6.49% (*n* = 12). Abscesses occurred in only one patient (0.54%). The total rate of major complications was 1.08% (*n* = 1). *Staphylococcus aureus* was the main microorganism causing the wound infections and the abscess. Data concerning the complications are presented in Table 3 and Figure 4.

### 3.4. Satisfaction Assessment

The satisfaction survey was filled out by 122 out of 185 patients (65.95%). Patients were asked to assess their satisfaction from “Very Dissatisfied” to “Very Satisfied” in five categories: (1) the general silhouette shape; (2) the aesthetics of the abdomen; (3) the shape of the buttocks; (4) the size of the buttocks; and (5) the projection of the buttocks. In each category, the majority of the patients were very satisfied. However, the satisfaction was the highest regarding the general silhouette shape and the aesthetics of the abdomen (very satisfied in 70.5% and 72.1%, respectively). Only 1.6% of the patients in each category stated that they were very dissatisfied. The results regarding patients’ satisfaction are demonstrated in Table 4.

## 4. Discussion

Buttock augmentation with fat grafting has gained significant popularity due to its natural-looking results and the fact that it involves both liposuction and buttock enhancement. However, gluteal augmentation involves another major technique to achieve the desired shape and size of the buttocks, mainly gluteal augmentation with implants. Both methods involve unique considerations, including the positioning of implants in different planes (such as submuscular, intramuscular, or subfascial) and the plane of fat graft injection (such as subcutaneously or intramuscularly). Recent reports have highlighted the effectiveness of the submuscular technique, which is associated with a lower risk of complications like fluid accumulation resulting from muscle fiber dissection or implant exposure/extrusion [12,13]. In a systematic review assessing the safety and efficacy of gluteal augmentation [14], the overall complication rate was found to be lower with autologous fat grafting (9.9%) compared to silicone buttock implants (21.6%). However, individuals with a slender physique and insufficient gluteal projection often have limited options and may choose gluteal implant surgery due to the lack of noticeable donor areas necessary for fat grafting. Hence, it is crucial to thoroughly assess each patient to determine the most suitable option that will yield the best aesthetic outcomes.

The procedural protocol for buttock augmentation with fat grafting offers numerous variations, with differences observed at each step of the procedure, including variations in fat harvesting, fat processing, and fat injection techniques. In the present study, we used liposuction using ultrasound energy, as we think there are numerous advantages to using this technique. One of the primary advantages of ultrasonic liposuction is its precision and selectivity in targeting fat cells. By utilizing ultrasound energy, ultrasonic liposuction can specifically break down fat cells while sparing surrounding tissues such as nerves, blood vessels, and connective tissue. This precision reduces the risk of inadvertent damage to adjacent structures, contributing to improved safety. Due to the decreased damage to the connective tissue, ultrasonic liposuction may improve skin tightening, which may be especially appealing to patients with mild to moderate skin laxity. Although different techniques are used for the fat processing step, Strong et al. [15] demonstrated no significant difference in retention between decantation, filtration, or centrifugation, as well as between donor sites.

The fat injection step is highly relevant due to its association with the most life-threatening complications, such as the formation of fat emboli [16]. Interestingly, the meta-analysis conducted by Conde-Green in 2016 [4] stated that, most commonly, the fat graft was injected into both the subcutaneous and intramuscular planes (46.7%), and only 20% of the studies reported injecting only into the subcutaneous plane [4]. It was also reported that complications occurred more frequently following intramuscular fat grafting (28.7%) than subcutaneous fat grafting (4.0%). Furthermore, numerous cases of deaths related to gluteal augmentation with fat have been presented in the literature, mainly due to the formation of macrofat embolisms [17]. A retrospective study from 2015 conducted by Cárdenas-Camarena et al. [18] reported 22 fatalities linked to pulmonary fat embolisms following gluteal fat grafting procedures in Mexico and Colombia, spanning a 15-year duration. They concluded that intramuscular gluteal fat injection is associated with mortality caused by damage to the gluteal vasculature, allowing the formation of macroscopic and microscopic fat embolisms. Due to the potentially life-threatening risks that are associated with intramuscular injections, more recent reports have stressed the importance of performing subcutaneous injections only. Cansancao et al. [17] performed a study based on reports from an expert opinion survey sent to and answered by members of the Brazilian Society of Plastic Surgery. One major highlight of the results of the study was that the risk of death was 16 times greater when fat was injected intramuscularly. In order to decrease the risk of life-threatening complications, they created a list of safety recommendations for performing gluteal fat grafting, with tips including injecting fat into the subcutaneous plane only and only injecting fat with the cannula in motion, amongst others. In the present study, we followed the majority of the recommendations on the list to ensure the highest level of safety for our patients (a modified list of safety recommendations for gluteal augmentation with fat grafting is presented in Table 5). However, these days, the gold standard for a safe injection is performing the injection with intraoperative ultrasound imaging. This can ensure that the injection process is solely performed in the subcutaneous plane and not intramuscularly. Nevertheless, we firmly believe that every plastic surgeon who performs gluteal augmentations with fat grafting should consider these recommendations because they are essential for ensuring patient safety, which should always be a top priority for any surgeon. This is especially relevant when performing this procedure, as it is associated with potentially life-threatening complications.

Although the formation of fat embolus is an extremely worrying potential complication, numerous, less severe complications occur more frequently. In the most recent systematic review conducted by Oregi et al. [7] on the safety profiles of implants and autologous fat grafting in gluteal augmentation, the complication rates associated with these procedures were analyzed based on the results in the literature. In this study, the general complication rate was calculated to be 13%, and the single most prevalent complication was seroma (6.9%). Interestingly, infections were the second most common complication, with a rate of 3.0%. Our retrospective analysis demonstrated ten cases of seromas (5.41%), two cases of minor infections (1.08%), and one case of abscess formation (0.54%). These rates correspond to the reports of other studies in the literature [4,7,19,20]. Luckily, the most frequently observed complications related to gluteal augmentation with fat grafting are uncomplicated to treat, are not life-threatening, and do not disrupt the overall aesthetic outcomes of the patient.

**Figure 5:**
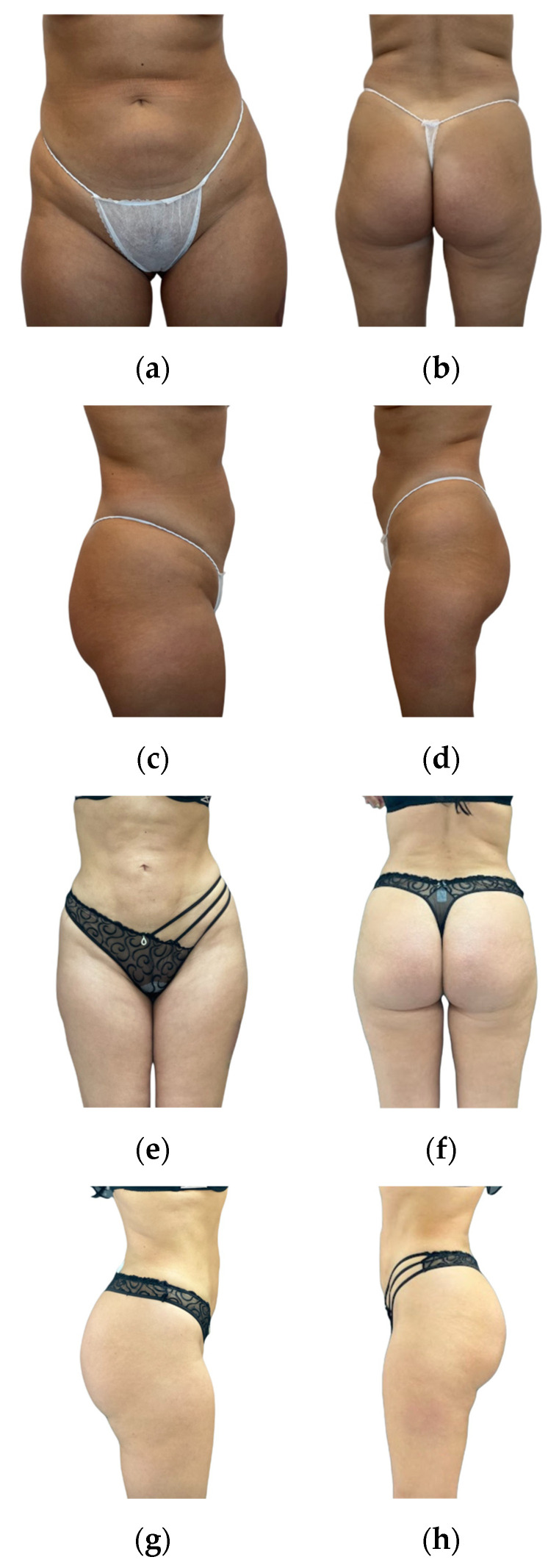
Results in a 33-year-old female patient 20 months after surgery (fat graft volume: 550 mL; height: 167 cm; and weight: 66 kg). (**a**–**d**) present the presurgical photos, whereas (**e**–**h**) presents the postsurgical photos.

**Figure 6:**
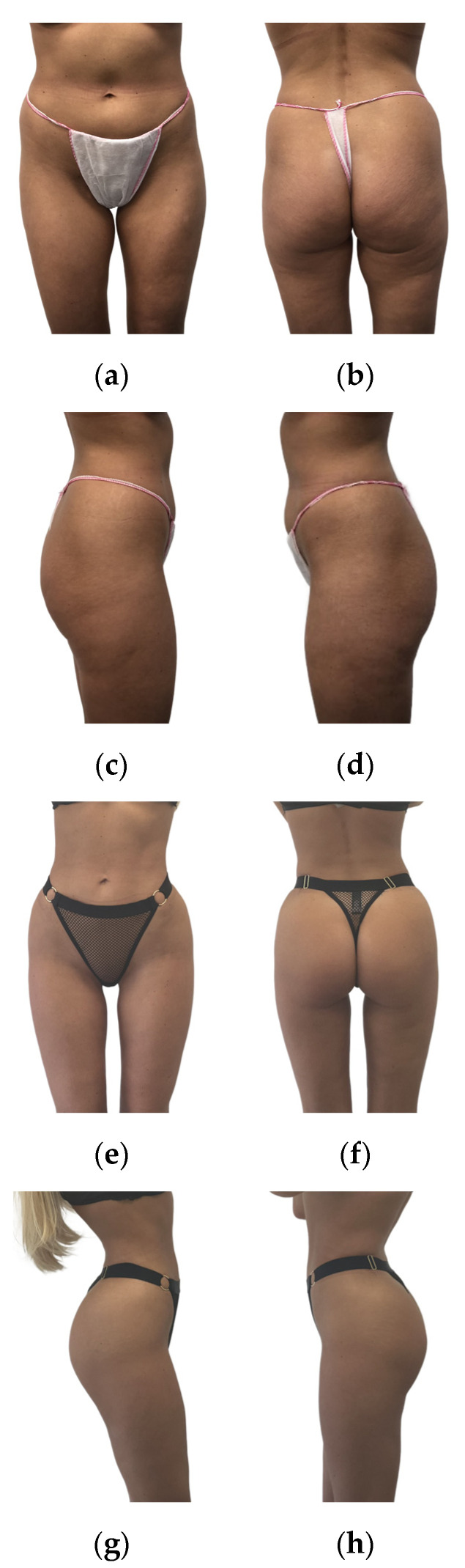
Results in a 36-year-old female patient 20 months after surgery (fat graft volume: 450 mL; height: 164 cm; and weight: 48 kg). (**a**–**d**) present the presurgical photos, whereas (**e**–**h**) presents the postsurgical photos.

Overall, we believe that gluteal augmentation with ultrasonic liposuction and fat grafting provides patients with satisfactory aesthetic results. Patient satisfaction, as revealed by our assessments, demonstrates positive aesthetic outcomes (postoperative outcomes are presented in Figure 5 and Figure 6). It is crucial to stress that the successful and secure application of this technique relies on careful patient selection, surgeon expertise, and commitment to safety protocols. Every surgeon performing gluteal augmentation with fat grafting needs to follow the appropriate safety recommendations to decrease the occurrence of potentially life-threatening complications, especially regarding the injection process being performed with intraoperative ultrasound imaging. When performed by experienced and well-trained surgeons, gluteal augmentation through ultrasonic liposuction and ultrasound-guided fat grafting is a reliable and secure way for patients to achieve their desired aesthetic enhancements.

The major limitation of the current study is the lack of a structured and standardized assessment of aesthetic outcomes using a certified scale. This presents difficulties in quantitatively evaluating and comparing aesthetic results among our patients. Moreover, the results regarding satisfaction after the procedure may not adequately portray the true satisfaction rates of our patient group, as only 65.95% of our patients filled out the satisfaction survey.

## 5. Conclusions

Achieving attractive aesthetic results following gluteal augmentation through fat graft transfer depends on maintaining a balanced approach between liposuction and precisely injecting fat into the buttocks. This approach aims to create a buttock with a pleasing size, shape, and projection that harmonizes with the patient’s overall silhouette. Based on our experience, ultrasonic liposuction and ultrasound-guided fat grafting for buttock augmentation are secure and dependable techniques for enhancing gluteal contours. Nevertheless, every precaution must be taken to minimize the risk of severe complications that could pose a danger to the patient’s life.

## Figures and Tables

**Table 1:** General characteristics of the 185 female patients included in the present study. IQR—interquartile range. kg—kilograms. cm—centimeters. BMI—Body Mass Index. mL—milliliters. *—the data relate to the volume of the fat graft injected unilaterally. Every patient received two equal volumes of fat grafts in each buttock.

Category	N	Mean	Standard Deviation	Median	Min	Max	IQR
Age	185	34.56	7.31	33.00	22.00	56.00	10.00
Weight (kg)		64.46	8.22	63.00	48.50	98.00	11.00
Height (cm)		167.39	5.82	168.00	152.00	188.00	6.00
BMI		23.00	2.62	22.57	17.58	34.31	3.26
Fat graft * (mL)		596.75	67.29	600.00	500.00	800.00	50.00

**Table 2:** Statistical results of the analysis of the correlation between the volume of the fat graft used and the patients’ age, weight, height, and BMI. Spearman’s rank correlation analysis was performed. A *p*-value lower than 0.05 was considered to be statistically significant. kg—kilograms. cm—centimeters. BMI—Body Mass Index. mL—milliliters. *—The data relate to the volume of the fat graft injected unilaterally. Every patient received two equal volumes of fat grafts in each buttock.

Category	Fat Graft * (mL)		
	N	R	*p*-Value
Age	185	0.13	0.09
Weight (kg)		0.55	0.00
Height (cm)		0.20	0.01
BMI		0.50	0.00

**Table 3:** Summary of the complications that occurred among the patients.

Severity of the Complication	Type of Complication	Number of Patients (N = 185)	Percentage	
Minor complications	Seroma	10	5.41%	6.49%
	Minor infections by the incision site	2	1.08%	
Major complications	Abscess	1	0.54%	1.08%

**Table 4:** Results regarding the patients’ satisfaction survey.

Category	Very Satisfied	Somewhat Satisfied	Somewhat Dissatisfied	Very Dissatisfied
The general silhouette shape	86 (70.5%)	26 (21.3%)	8 (6.6%)	2 (1.6%)
The aesthetics of the abdomen	88 (72.1%)	21 (17.2%)	11 (9.0%)	2 (1.6%)
The shape of the buttocks	82 (67.2%)	32 (26.2%)	6 (4.9%)	2 (1.6%)
The size of the buttocks	68 (55.7%)	48 (39.3%)	4 (3.3%)	2 (1.6%)
The projection of the buttocks	67 (54.9%)	47 (38.5%)	6 (4.9%)	2 (1.6%)

**Table 5:** Modified list of the safety recommendations presented by Cansancao et al. (2019) [17].

**Safety recommendations for performing gluteal fat grafting**
Ensure fat injection occurs only in the subcutaneous layer by utilizing intraoperative ultrasound imaging
Carefully select suitable patients for the procedure
Acquire expertise in the procedure and familiarize yourself with gluteal anatomy
Exercise caution when considering high-volume fat grafting
Implement effective clinical management techniques
Use a closed system for both fat harvesting and injection
Maintain continuous cannula movement during the fat injection stage
Utilize a larger diameter cannula for fat injection
Administer prophylactic antibiotics

## Data Availability

The data that support the findings of this study are available from the corresponding author upon reasonable request.

## Supplementary Materials

The following supporting information can be downloaded at: https://www.mdpi.com/article/10.3390/jcm13061526/s1, Video S1: Video illustrating the surgical technique of gluteal augmentation with ultrasonic liposuction and fat grafting.
